# Cells that survive acute murine SARS-CoV-2 infection are detected nearly exclusively in the respiratory tract

**DOI:** 10.1172/JCI172659

**Published:** 2023-11-15

**Authors:** Ruangang Pan, David K. Meyerholz, Stanley Perlman

**Affiliations:** 1 Department of Microbiology and Immunology,; 2 Department of Pathology, and; 3 Department of Pediatrics, University of Iowa, Iowa City, Iowa, USA.

**Keywords:** COVID-19, Infectious disease, Molecular biology, Mouse models

**To the Editor:** Long-term sequelae of SARS-CoV-2 infection, referred to as postacute sequelae of COVID-19 (PASC), involve many organs, including the cardiovascular, pulmonary, gastrointestinal, and neurological systems. The basis of PASC is not well understood, but one possible explanation is that the persistence of infectious virus or viral RNA or protein contributes to PASC. This is difficult to assess in patients because human tissue can only usually be assessed for virus or viral products at autopsy or by biopsy. Furthermore, it is not known if some SARS-CoV-2–infected cells survive the acute infection and contribute to long-term sequelae. To address this issue in mice, we used a lineage-tracing approach that allows longitudinal tracking of previously infected cells over several months (1, 2).

For this purpose, two recombinant SARS-CoV-2 expressing cre recombinase and Venus fluorescent proteins were engineered using a previously described BAC reverse genetics system (3) (see Supplemental Methods; supplemental material available online with this article; https://doi.org/10.1172/JCI172659DS1). We used these viruses to infect mice expressing a loxP-flanked STOP cassette in the tdTomato gene driven by the Rosa26 promoter (Ai9 mice). Venus and Cre were linked by a 2A peptide (Venus-2a-Cre [V2C]), which after autocleavage resulted in the release of the two proteins (Figure 1A). V2C was inserted into ancestral SARS-CoV-2 (Wuhan-Hu-1; rSARS2-WH-V2C), which cannot infect laboratory mice but can infect hACE2 transgenic (K18-hACE2) mice (4), and into mouse-adapted SARS2-N501Y_MA30_ (rSARS2-MA30-V2C), which infects all strains of laboratory mice (5). Venus facilitated identification of acutely infected cells (Figure 1, B–D), while Cre expression resulted in permanent tdTomato labeling of cells that survive the acute infection (Figure 1, B and C). rSARS2-WH-V2C and rSARS2-MA30-V2C were as virulent as control viruses in K18-hACE2/Ai9 and Ai9 mice, respectively (Figure 1E and Supplemental Table 1). Pathological changes in the lungs (degree of hemorrhage, edema, perivascular infiltrates) were consistent with virulence (Figure 1F and Supplemental Methods). Venus expression was detected between 1 and 5 days after infection (dpi), with peak expression found at 2 dpi. tdTomato cells were detected no earlier than 7 dpi. We detected no cells dually labeled with Venus and tdTomato after infection.

We used this system to assess infection of several organs at 2 dpi as well as at 20 and 60 dpi, when infectious virus was no longer detected. For both experimental systems, we analyzed at least 5 mice and 10–20 slides per mouse. We found virus predominantly, if not solely, in the respiratory tract of all mice, including the lungs and nasal cavity (Figure 1G). Analysis of mice at 2 dpi showed occasional virus-infected cells in the intestine but not in the brain, heart, spleen, or liver (Supplemental Table 2). The intestine was the only extrapulmonary organ in which infected cells could be detected at 2 dpi (Figure 1G and Supplemental Table 2). In the intestine, a common site of SARS-CoV-2 infection in patients (6), virus was detected in occasional, isolated cells, suggesting that infection was nonproductive or, if productive, spread to adjacent cells was inefficient.

Consistent with the analyses of acutely infected mice, cells that survived the initial infection were nearly solely found in the respiratory tract at both 20 and 60 dpi (Figure 1, H and I). The lungs, specifically the lung parenchyma, harbored the majority of the surviving cells, while only a few were detected in the nasal cavity and distal airway. Surviving cells in the nasal cavity and lung bronchiole were detected at much lower levels than during the acute phase of the infection (compare Figure 1G with Figure 1H for rSARS2-MA30-V2C–infected Ai9 mice), suggesting that only a small fraction of these cells survived the acute infection. The majority of the surviving cells were located in the alveoli (Figure 1H). Analysis of lung sections at 60 dpi revealed a further decline in numbers of surviving cells compared with those at 20 dpi (Figure 1J). Notably, some of the surviving cells in the alveoli displayed increased branching at 60 dpi relative to 20 dpi (Figure 1H vs. Figure 1I), consistent with lung regeneration. No surviving cells were detected in the intestine (Supplemental Table 3), indicating that none of the infected cells survived the acute infection or they underwent homeostatic turnover. Results were virtually identical in rSARS2-WH-V2C–infected K18-hACE2/Ai9 and rSARS2-MA30-V2C–infected Ai9 mice, with the exception that rare surviving cells were identified in the brains and hearts of the former (Figure 1K and Supplemental Table 3).

All together, this study demonstrates that cells that were previously infected with SARS-CoV-2 survived for extended periods of time only in the respiratory tract, with no evidence of significant survival in other organs. These results, which require validation in humans, suggest that the host response and not persistent virus infection is most important for extrapulmonary sequelae.

## Supplementary Material

Supplemental data

Supporting data values

## Figures and Tables

**Figure 1:**
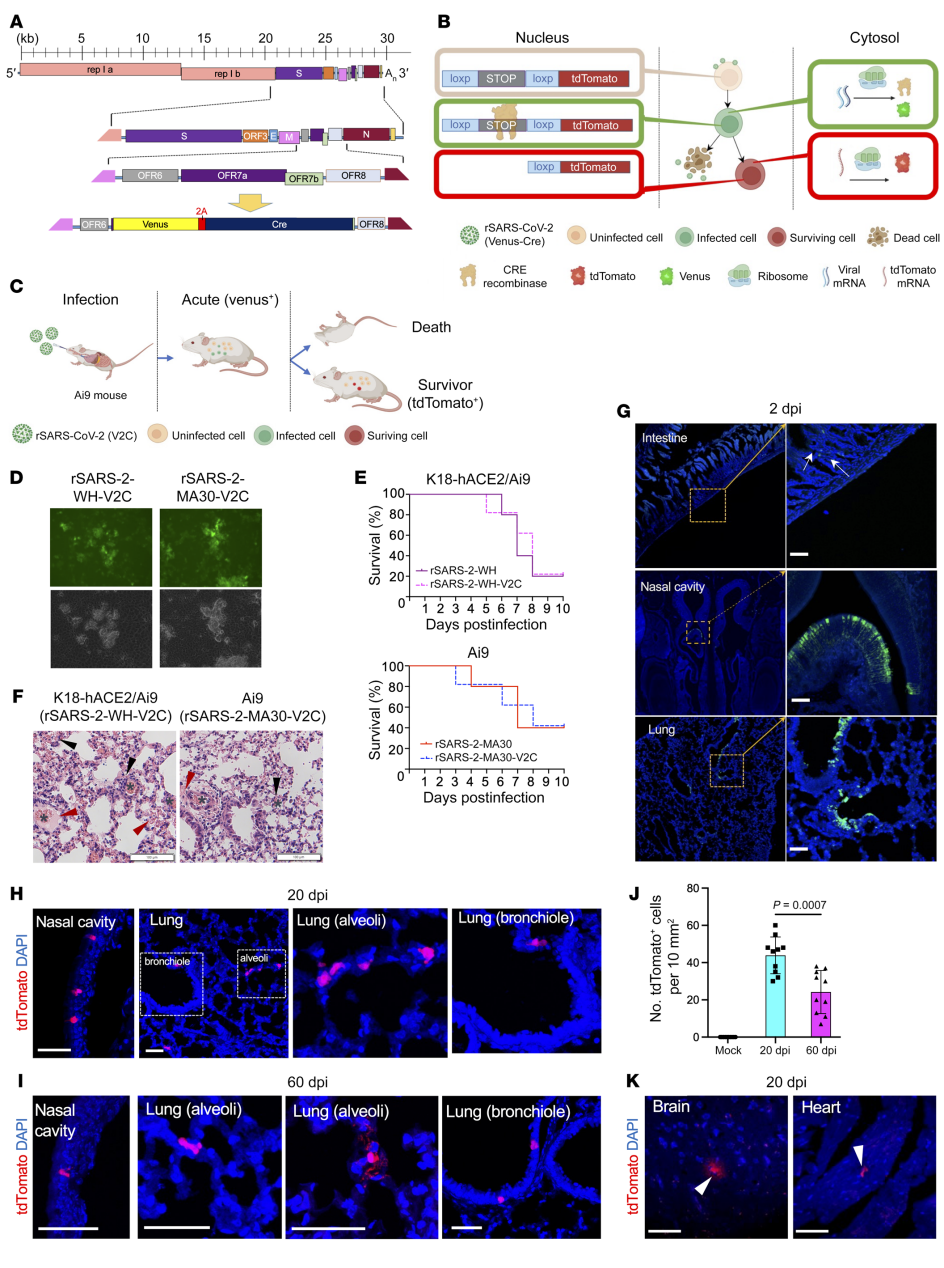
SARS-CoV-2–infected cells survive acute infection and persist in the respiratory tract. (**A**) Schematic showing construction of recombinant SARS-CoV-2 harboring Venus and Cre in place of the ORF7a gene. (**B**) Schematic showing Venus and Cre expression. (**C**) Schematic of lineage tracing using recombinant viruses. Infected cells are Venus^+^ (green) at early times after infection, while surviving cells are tdTomato^+^ (red). (**D**) Infected VeroE6 cells (MOI = 0.01) were analyzed for GFP expression (top) and by bright-field microscopy (bottom) at 48 hours after infection (original magnification, ×20). (**E**) Mice were infected with the indicated viruses (K18-hACE2/Ai9 mice, 3,000 PFU rSARS-2-WH-V2C; Ai9 mice, 3,000 PFU rSARS-2-MA30-V2C) and monitored for survival (*n* = 5 mice per group). (**F**) Pathological changes in lungs of mice infected with rSARS-2-WH-V2C or rSARS-2-MA30-V2C at 4 dpi. Asterisks, hemorrhage; red arrowheads, edema; black arrowheads, perivascular infiltrates. Scale bar: 100 μm. (**G**) Sections from the indicated organs harvested from rSARS2-MA_30_-V2C–infected Ai9 mice were analyzed for Venus expression at 2 dpi. Venus^+^ cells were observed in the intestine (white arrows), nasal cavity, and lung. Scale bar: 50 μm. (**H** and **I**) Sections from the nasal cavities and lungs of rSARS2-MA_30_-V2C–infected Ai9 mice were analyzed for tdTomato expression at 20 (**H**) and 60 dpi (**I**). (**J**) Summary of numbers of tdTomato^+^ cells in the lungs of rSARS2-MA_30_-V2C–infected Ai9 mice. Each group contain 5 animals (*n* > 3 sections per animal were analyzed). (**K**) Rare tdTomato^+^ cells were found in the brains and hearts of rSARS2-WH-V2C–infected K18-hACE2/Ai9 mice at 20 dpi. Arrowheads indicate surviving cells. In **D**, **F**, and **G**–**K** images are representative of 2 independent experiments (5 mice/group, *n* = 4–10 slides per mouse). Higher magnification images of selected areas are shown in insets (**G** and **H**). Scale bars: 50 μm (**G**–**K**).

## Version 1 11/15/2023

Electronic publication
