# Supplementary material for: Cells that survive acute murine SARS-CoV-2 infection are detected nearly exclusively in the respiratory tract
Source: J Clin Invest. 2023 Nov 15;133(22):e172659. doi: 10.1172/JCI172659 (PMC10645383; doi:10.1172/JCI172659)
Supplement: Supplemental data [file jci-133-172659-s232.pdf]

Supplemental Material

Acknowledgements: This study was supported in part by grants from the NIH (PO1 AI060699 and RO1 AI129269). The authors acknowledge use of the University of Iowa's Central Microscopy Research Facility.

Materials and Materials

Mice

Specific-pathogen-free C57BL/6 mice were purchased from Charles River Laboratories. B6.Cg-*Gt(ROSA)26Sor*$^{tm14(CAG\text{-}tdTomato)Hze}$/J (Ai9-tdTomato) mice and 034860-B6.Cg-Tg(K18-ACE2)2Prlman/J (K18-transgenic mice expressing hACE2, K18-hACE2) were purchased from Jackson Laboratories. Eight- to twelve-week-old mice were intranasally inoculated with the indicated viruses (**Table 1**) after isoflurane anesthesia. After virus infection, mice were monitored and weighed daily. All experiments with SARS-CoV-2 were performed in a Biosafety Level 3 (BSL3) laboratory at the University of Iowa. All animal studies were approved by the University of Iowa Animal Care and Use Committee and meet stipulations of the Guide for the Care and Use of Laboratory Animals.

Generation of SARS-CoV-2 or SARS-CoV-2-MA30 encoding the Venus and cre proteins.

p-BAC SARS-CoV-2 carrying the sequence of the isolate Wuhan-Hu-1 (WT-SARS-CoV-2 BAC) was constructed as previously described (1). p-BAC-SARS-CoV-2 with

mouse-adapted mutations was generated as previously described (2). pBAC-SARS-CoV-2 and mouse-adapted pBAC-SARS-CoV-2-MA30 encoding Venus-2a-Cre protein were engineered using previously described methods(3). Primers used to introduce Venus-2a-CRE into pBAC-SARS-CoV-2 and pBAC-SARS-CoV-2-MA30 are as follows:

Forward primer for the GalK fragment:

ATTAAACGAACATGAAAATTATTCTTTTCTTGGCACTGATAACACTCGCTCCTGTTGACAATTAATCATCG

Reverse primer for the GalK fragment:

GGAATAGCAGAAAGGCTAAAAAGCACAAATAGAAGTCAATTAATGAAAGTTCAACTCAGCAAAAGTTCGATTTA

Forward primer for the Venus-2a-Cre fragment:

ATTAAACGAACATGAAAATTATTCTTTTCTTGGCACTGATAACACTCGCTGTGAGCAAGGGCGAGGAGC

Reverse primer for the Venus-2a-Cre fragment:

TCTGTCTTTCTTTTGAGTGTGAAGCAAAGTGTTATAAACACTATTGCCGCGTCGCCGTCCAGCAGTC

All mutations and the Venus-2a-Cre insertion were confirmed by sequencing.

Rescue of SARS-CoV-2 and SARS-CoV-2-MA30 encoding the Venus and Cre proteins.

Confluent monolayers of Vero E6 cells overexpressing hACE2 and TMPRSS2 (A2T2 Cells) (10^6 cells per well, six-well plates) were transfected with 2.0 ug per wells of SARS-CoV-2 BAC using Lipofectamine 3000. Cells were monitored daily for cytopathic effects (CPEs). Cultures were collected when CPE was >50%, and frozen at -80 °C. Recombinant viruses were propagated in A2T2 cells in DMEM supplemented with 10% fetal bovine serum (FBS). Cultures were collected when CPE was >50%, and frozen at -80 °C, Virus titers were determined by plaque assay.

Plaque assay

12-well plates of Vero E6 cells were inoculated at 37 °C and gently rocked every 15 min for 1 h. After removing the inocula, plates were overlaid with 0.6% agarose containing 5% FBS. After 3 days, plates were fixed in 10% PFA for over 60 minutes, then the overlays were removed, and plaques were visualized by staining with 0.1% crystal violet for 20 minutes. Viral titers were quantified as PFU per ml.

Tissue processing.

Routine mouse perfusion procedures were performed. Briefly, mice were anesthetized with a final concentration of 100 mg kg^{-1} ketamine in accordance with Institutional Animal Care and Use Committee guidelines. Mice were then transcardially perfused with PBS followed by freshly prepared 10% PFA in PBS. Lungs, nasal cavity, heart and brain were post-fixed in 10% PFA in PBS overnight at 4 °C. After 24 hours, organs (lungs, heart and brain) were cryoprotected by

immersion in 10% sucrose for 30 minutes, followed by immersion in 20% sucrose overnight. Organs (lungs, heart and brain) were subsequently infiltrated in 30% sucrose and kept at 4°C overnight. Subsequently, organs (lungs, heart and brain) were snap-frozen in tissue freezing media (OCT, Fisher HealthCare). 8-10 μM sections were obtained using a HM525 cryostat (Thermo Fisher Scientific) and stored at -80°C.

The nasal cavity was fixed in zinc formalin, decalcified using EDTA, and embedded in OCT before sectioning on a cryostat.

For lung histology, formaldehyde-fixed, paraffin-embedded tissue sections (4 mm each) were stained with H&E.

Data availability. Values for all data points in graphs are reported in the Supporting data values file. Data are available upon request.

Statistics. Data were analyzed using two-tailed Student *t*-tests. A P value of <0.05 was considered significant. Mann-Whitney *U* tests were used to analyze differences in means when samples were nonparametric. Results are represented as means ± SEM. **P*≤0.05, ** *P*≤0.01 and *** *P*≤0.001.

Author Contributions. RP, SP designed the experiments. RP performed the experiments. RP, DM, SP analyzed the results. RP and SP wrote the first draft of the manuscript. All of the authors contributed to the final draft.

Reference:

1. Almazán F, Galán C, and Enjuanes L. Engineering infectious cDNAs of coronavirus as bacterial artificial chromosomes. *Methods Mol Biol.* 2008;454:275-91.
2. Wong L-YR, Zheng J, Wilhelmsen K, Li K, Ortiz ME, Schnicker NJ, et al. Eicosanoid signalling blockade protects middle-aged mice from severe COVID-19. *Nature.* 2022;605(7908):146-51.
3. Fehr AR, Channappanavar R, Jankevicius G, Fett C, Zhao J, Athmer J, et al. The Conserved Coronavirus Macrodomain Promotes Virulence and Suppresses the Innate Immune Response during Severe Acute Respiratory Syndrome Coronavirus Infection. *mBio.* 2016;7(6).

Supplemental Tables

Table 1. Mouse and virus strains

Mouse strain	K18-hACE2 Tg x Ai9 (K18-hACE2/Ai9)	Ai9 mice
Virus	rSARS-2-WH-Cre-Venus (rSARS2-WH-V2C)	rSARS-2-MA30-Cre-Venus (rSARS-2-MA30-V2C)
Abbreviation	K18-hACE2-WH	Ai9-MA30
Lethality	>70% at 3000 PFU/mouse	~40-60% at 3000 PFU/mouse

Table 2. Comparison of cell tropism during acute infection (2dpi, Venus+)

Mouse strain		K18-hACE2/Ai9	Ai9 mice
Virus		rSARS2-WH-V2C	rSARS-2-MA30-V2C
Organ	Brain	-	-
	Nasal cavity	***	***
	Lung	****	****
	Heart	-	-
	Spleen	-	-
	Liver	-	-
	Intestine	**	**

Table 3. Comparison of surviving cell locations (20 dpi, tdTomato+)

Mouse strain		K18-hACE2/Ai9	Ai9 mice
Virus		rSARS2-WH-V2C	rSARS-2-MA30-V2C
Organ	Brain	*	-
	Nasal cavity	**	**
	Lung	***	***
	Heart	*	-
	Spleen	-	-
	Liver	-	-
	Intestine	-	-

Cells were identified post-infection in mice, and their numbers were indicated based on two fluorescence markers: Venus+ (indicative of infected cells at 2 days post-infection) and tdTomato+ (indicative of surviving cells at 20 days post-infection). The asterisks represent the range of surviving cell counts:

* Surviving cells in the **heart** was found in only 1 out of 5 mice. For this particular mouse, no more than 1 surviving cell was observed across 5 slides.
Surviving cells in the **brain** were found in 3 out of 5 mice, no more than 5 surviving cells were observed across 5 slides for each mouse.

** 5-20 cells/slide, indicated cells were observed in all mice

*** 20-100 cells/slide, indicated cells were observed in all mice

**** more than 100 cells/slide, indicated cells were observed in all mice
